# Repeatability of echocardiographic parameters to evaluate the hemodynamic relevance of patent ductus arteriosus in preterm infants: a prospective observational study

**DOI:** 10.1186/s12887-016-0552-7

**Published:** 2016-01-26

**Authors:** Christoph E. Schwarz, Antonio Preusche, Winfried Baden, Christian F. Poets, Axel R. Franz

**Affiliations:** Department of Neonatology, University Children’s Hospital of Tuebingen, University of Tuebingen, Calwerstr. 7, 72076 Tuebingen, Germany; Department of Pediatric Cardiology, University Children’s Hospital of Tuebingen, University of Tuebingen, Tuebingen, Germany; Center for Pediatric Clinical Studies, University Children’s Hospital of Tuebingen, University of Tuebingen, Tuebingen, Germany

**Keywords:** Reproducibility, Doppler-ultrasound, Inter-observer

## Abstract

**Background:**

The hemodynamically relevant patent ductus arteriosus in preterm infants is not well defined. Different clinical and echocardiographic parameters are used and the diagnostic accuracy is unknown because of the lack of a gold standard definition. Our study evaluates the inter-observer repeatability of echocardiographic and Doppler-ultrasound parameters.

**Methods:**

This prospective observational study included 19 very low birth weight preterm infants (median [interquartile range]: gestational age 28.0 (28.0–29.0) weeks, birth weight 1130 (905–1321) g, postnatal age at measurement 8.7 (4.8–23.5) d) with a clinical suspicion of ductal patency in whom 27 repeated echocardiographic and Doppler-ultrasound examinations were performed within 30 min by 2 of 3 independent observers (54 measurements overall). The repeatability index (=2 times the standard deviation of the differences/mean of all measurements) according to Bland and Altman was used to assess repeatability of different parameters.

**Results:**

The repeatability indices of the echocardiographic parameters (left Atrium-to-Aortic root-ratio, diameter of the patent ductus arteriosus at its narrowest part, the left-ventricular-preejection-period-to-ejection-time-ratio and the ratio of the velocity time integrals in the large vessels were 16, 21, 23 and 26 % respectively. The repeatability indices of Doppler-ultrasound measurements (resistance index in celiac artery and anterior cerebral artery) were 11 and 14 %, respectively.

**Conclusions:**

The inter-observer repeatability of all echocardiographic parameters was poor compared to that of resistance indices in peripheral vessels. Therefore, interventions for ductal patency should be indicated based on averaged repeated rather than single measurements, especially when measured values are close to their cut-off value - both in clinical routine and for study purposes.

## Background

The patent ductus arteriosus (PDA) in preterm infants is associated with increased mortality and morbidity [1–7]. However, there is little evidence as to which parameters define a PDA that requires treatment. Zonnenberg and de Waal showed that, besides clinical parameters, echocardiographic and Doppler-ultrasound measurements are used to evaluate the magnitude and clinical relevance of the left-to-right shunt through a PDA, and hence the need for treatment: In a systematic review of 67 randomised controlled trials (RCTs) they described the following most frequently used parameters and applied cut-off values: Left-atrium-to-aortic-root-ratio (LA/Ao-ratio) used in 34 trials, median cut-off >1.3 (range: 1.15–1.70); diastolic reverse flow in peripheral vessels (21 trials); and PDA-diameter (8 trials), cut-off >1.5 (1.5–2.0) mm [8]. McNamara and Sehgal suggested a scoring system including clinical and echocardiographic criteria to define hrPDA [9]. The echocardiographic part of this staging seems to be predictive for neonatal morbidity and can serve as a guide to clinical decisions [10], whereas the clinical criteria comprise unspecific respiratory signs. Prospective data suggesting that application of the echocardiographic parameters summarized by Zonnenberg and de Waal or the score by McNamara and Sehgal results in improved outcome is lacking. However, recent retrospective data suggest that echocardiographic screening for PDA within the first 3 postnatal days may reduce mortality in infants born at <29 weeks gestation [11].

To inform future studies and clinical guidelines on PDA treatment, this study aimed to evaluate the inter-observer repeatability of echocardiographic and Doppler-ultrasound parameters, which are frequently determined to assess the need for PDA treatment.

## Methods

This prospective observational cohort study was approved by the research ethics committee at the facutly of medicine and the university hospital of the Eberhard Karls University Tuebingen and written informed parental consent obtained. To assess the repeatability of echocardiographic parameters commonly used to determine the magnitude of the left-to-right shunt, a convenience sample of preterm infants with suspected PDA was analysed. Inclusion criteria were: birth weight ≤1500 g and clinical suspicion of PDA such as cardiac murmur, bounding pulses, ventilator dependency and increased oxygen demand. Syndromal anomalies and congenital heart defects except persisting foramen ovale or atrial septal defect were exclusion criteria. The period of recruitment was between June 2012 and May 2013 at the Department of Neonatology, University Children’s Hospital of Tuebingen, University of Tuebingen, Germany.

Within 30 min (to minimize fluctuations in hemodynamic status), 2 of 3 investigators (with more than 20, 10, or 3 years, respectively, of experience in neonatal echocardiography, everyday skills or every week, respectively, 2 were board certified paediatric cardiologists, one investigator is attending physician at the NICU) prospectively and independently performed repeated echocardiographic and Doppler-sonographic measurements including the following parameters:

LA/Ao-ratio [12]; resistance index (RI) in celiac artery (CA) [13] and anterior cerebral artery (ACA) [14]; diameter of the PDA at its narrowest part [15]; the left-ventricular-preejection-period-to-ejection-time-ratio, calculated by including 3–4 cardiac cycles (LVPEP/LVET) [16]; and the ratio of the velocity time integrals in the large vessels (VTI_Ao/VTI_Pa). The VTI_Ao was measured from an apical-5-chamber-view, the VTI_Pa was measured in a parasternal short axis calculated automatically with built-in software. We assumed that, in the absence of congenital heart defects, this ratio correlates with the ductal left-to-right shunt. The PDA-diameter was measured at its narrowest part (identified via colour Doppler) and measured in B-Mode to avoid the influence of gain-settings on the PDA-width if assessed on colour Doppler images.

All measurements were done with a Toshiba “Aplio” using a 6.5 MHz phased array transducer.

Statistical analyses involved repeatability coefficient (RepC = 2 times the standard deviation of the differences) and repeatability index (RepI = RepC/mean of all measurements) according to Bland and Altman [17] and a confidence-step-analysis (CSA) [18]. The RepC represents the upper limit of the 95 % confidence interval of the absolute differences between two measurements performed by two independent observers. The RepI describes the relation between RepC and the mean value of the measurements. This allows comparison of repeatability between different measures. A high CSA value (CSA = difference between lowest and highest value / RepC) indicates that differences between low and high values in this parameter observed in a given population are unlikely related to inter-/intra-observer variability, whereas a CSA of ≤1 indicates that observed differences between low and high values in this population are likely due to inter-/intra-observer variability.

For unexpected RepI differences, 95 % confidence intervals (CI) were exploratorily calculated post-hoc and differences between echo-parameters classified as ‘significant’ if these 95 %-CI did not overlap.

Data are shown as median (interquartile range).

## Results

Twenty-seven repeated measurements were performed in 19 preterm infants. One infant with a birth weight of 1550 g was included inadvertently due to a weight at the time of measurement of 1465 g.

Gestational age at birth was 28.0 (28.0–29.0) weeks, birth weight 1130 (905–1321) g, postnatal age at measurement 8.7 (4.8–23.5) d and weight at the time of measurements 1243 (1024–1528) g. The mean difference in time between the first measurements of the repeated echocardiographic examinations was 12 min with a standard deviation (SD) of 4 min. The mean heart rate while recording left ventricular time intervals during all examinations was 167/min, and mean heart rate difference between repeated examinations was 2/min with a SD of 10/min. Arterial oxygen saturation was targeted at 90–95 % if on supplemental oxygen. Supplemental oxygen was necessary in 8 patients (respiratory support: 4 intubated and ventilated, 4 on binasal CPAP). Of 19 infants in room air, 5 were without respiratory support and 14 on binasal CPAP. No infant required catecholamines, and 3 had indomethacin within 24 h prior to measurements (0.1/0.2/0.4 mg/kg bodyweight/day, respectively).

A left-to-right shunt was identified by colour-Doppler-ultrasound in 15/27 measurements. PDA-diameter at the narrowest part could rarely be measured by both investigators (n = 6) because of difficulties visualizing the PDA in its complete course in B-mode (not colour Doppler-mode). The results are presented in Table 1.

**Table 1:** Repeatability Index (RepI), Repeatability Coefficient (RepC) and Confidence-Step-Analysis (CSA) values for Echocardiographic Parameters in Preterm Infants with Suspected Patent Ductus Arteriosus

Parameter	N	CSA	Repeatability Coefficient		Repeatability Index	
			RepC	95 % CI	RepI [%]	95 % CI
RI_CA	23	6.5	0.09	0.07–0.13	11	9–17*
RI_ACA	23	4.1	0.11	0.08–0.16	14	11–20**
LA/Ao	23	4.9	0.23	0.17–0.33	16	12–23
LVPEP/LVET	27	3.3	0.08	0.06–0.11	23	18–32
VTI_Ao/VTI_Pa	23	8.2	0.28	0.21–0.40	26	20–38
PDA - diameter	6	7.6	0.28	0.15–1.47	21	12–112

RI (resistance index) in CA (celiac artery) and ACA (anterior cerebral artery), LA/Ao-ratio (Left-atrium-to-aortic-root-ratio), LVPEP/LVET (left-ventricular-preejection-period-to-ejection-time-ratio), VTI_Ao/VTI_Pa (ratio of the velocity time integrals in the large vessels) and PDA diameter (patent ductus arteriosus); 95 % CI (95 % confidence interval) significantly smaller than RepI of LVPEP/LVET and VTI_Ao/VTI_Pa marked with “ * ”, “significantly” smaller than RepI of VTI_Ao/VTI_Pa marked with “ ** ”

## Discussion

In general, a good diagnostic parameter can easily and quickly be determined and has high repeatability, sensitivity and specificity. Neonatal echocardiography can be performed easily and quickly to determine the need for treatment in preterm infants with PDA, however, the diagnostic accuracy is unknown because of the lack of a gold standard definition of a hrPDA.

This work on the largest cohort reported to date shows that repeatability of neonatal echocardiographic and Doppler-ultrasound parameters in preterm infants with suspected PDA is far from optimal. This is not due to a lack of expertise because our results are in the range of those few reports that previously addressed the issue of the repeatability of echocardiographic parameters in smaller cohorts (Table 2) [18–22]. However, as summarised in Table 2, of the parameters elected here, only the RI_ACA has previously been addressed in a repeatability study.

**Table 2:** Repeatability Index (RepI) of Echocardiographic Parameters in Paediatric Patients According to the Literature, [18–22]

author, year	N	Gestational age/Postnatal age		Birthweight		Parameter	RepI
		Mean (SD)/Median	Range	Mean (SD)/Median	Range		
Groves, 2008	9	28 w	27–30 w	1250 g	910–1900 g	diameter Aorta descendens	31 %
						VTI Aorta descendens	57 %
Skinner, 1996	26	34 w	26–40 w	2406 g	975–4480 g	PDA Vmax sys.	28 %
						PDA Vmean	36 %
Hudson, 1990	20	NR	27–43 w	NR	2380–4020 g	Ao Leading Edge to Leading Edge	10 %
Moorthy, 1990	12	“preterm”		NR		RI_ACA	20 %
van Dijk, 1996	44	8.4 (4.7) y		NR		RVPEP	17 %
						RVET	57 %

*Abbreviations*: *VTI* velocity time integral, *PDA* patent ductus arteriosus, *Ao* Diameter Aorta, *RI_ACA* resistance index in anterior cerebral artery, *RVPEP* right ventricular preejection period, *RVET* right ventricular ejection time, *w* weeks, *y* years, *NR* not reported

In fact, our protocol simulated a “best case scenario”, as it evaluated repeated measurements by experienced investigators using the same ultrasound device within a short time interval on the same patient. Our study adds that the concerns regarding repeatability raised in the 1990s [18–22] are still relevant today despite improved ultrasound technology. Nevertheless, knowledge about the poor repeatability has not yet been taken into account in clinical treatment guidelines or current study protocols. The comparability and generalizability of results of data on echocardiography-guided PDA treatment are limited because of differences in the parameters applied and the poor reproducibility of all these parameters.

A large number of echocardiographic and Doppler-ultrasound parameters are used to quantify left-to-right shunt through, and hemodynamic relevance of, a PDA (summarised in [8]). These may include ductal flow pattern and velocity, absent or reverse diastolic flow in superior mesenteric artery, diastolic flow velocity in left pulmonary artery, reverse flow in descending aorta, and LVO/SVC-flow ratio (left ventricular output/superior vena cava-flow ratio). Some of these parameters may include redundant information [23], others, such as LVO/SVC-flow ratio, may not be trivial to measure, because of the complex cross sectional area of the SVC. Our selection of parameters reflects local preferences and was limited to reduce examination time and hence study-driven burden on the infants and subsequent fluctuations of their hemodynamic status in time.

In general, precision of a measurement with poor repeatability can be increased by averaging results of repeated measurements. In the context of this study, the effect of averaging measurements was cut-off dependent: Choosing, for example, cut-offs of >1.15, 1.3, 1.5, and 1.7 for the LA/Ao-ratio as the most frequently used parameter (i.e., cut-offs previously reported [8]) would result in *n* = 22, 16, 10 and 5, respectively, of the 27 episodes with at least one single-observer-measurement above the cut-off. In contrast, if only the mean of 2 measurements were considered, LA/Ao would have been above the cut-off in 20, 15, 8 and 2 episodes, indicating that in 4–11 % of cases a treatment decision based on LA/Ao-ratio would have been changed by averaging results of only 2 repeated measurements.

**Fig. 1:**
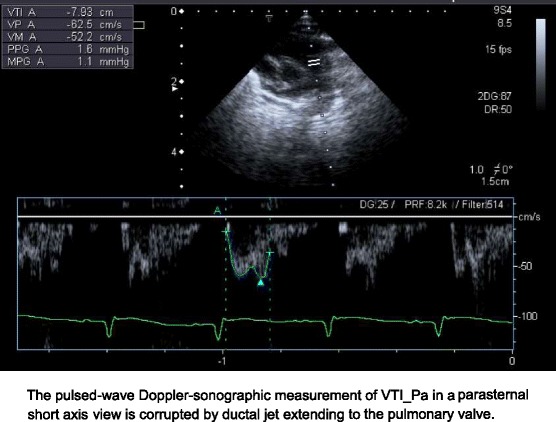
Measurement of VTI_Pa in a parasternal short axis view. The pulsed-wave Doppler-sonographic measurement of VTI_Pa in a parasternal short axis view is corrupted by ductal jet extending to the pulmonary valve

Before embarking on this study, we assumed that the VTI_Ao/VTI_Pa-ratio might be another easily determined parameter suitable for quantifying ductal left-to-right shunt. Unfortunately, repeatability was similarly poor, presumably because this parameter required measurements in two different views (parasternal short axis and apical 5-chamber view) and VTI_PA was corrupted by the ductal jet (Fig. 1). It is also important to note that VTI_Ao/VTI_Pa-ratio may not accurately reflect the degree of shunt through a PDA because of inter-atrial shunting which is commonly observed in VLBW infants just like in our cohort (only 1 out of 19 infants had no inter-atrial shunting, no infant had a ventricular septal defect). This latter limitation also applies to more commonly used parameters such as the LA/Ao ratio. Furthermore, the assumption underlying the determination of VTI_Ao/VTI_Pa-ratio that the cross-sectional areas of P- and Ao-valve are similar may not applicable to all infants. However, despite poor repeatability, VTI_Ao/VTI_Pa had a high CSA-value, indicating a high potential in identifying inter-individual differences and consequently permitting accurate classification (Table 1). Similarly, determination of the PDA-diameter was challenging, because it requires visualisation of the PDA from the aorta to the pulmonary artery.

A limitation of our study is that extremely immature preterm infants with the highest risk of PDA are under-represented because we were hesitating to subject these most vulnerable infants during their first postnatal days to repeated measurements. Future studies need to assess intra-observer repeatability.

## Conclusions

The repeatability of echocardiographic parameters to evaluate ductal left-to-right shunt is poor. The highest repeatability was achieved by RIs in ACA and CA. This has implications for clinical practice as well as the design of future studies on PDA treatment. In both settings, repeated measurements and averaging of results should be implemented, especially when measured values are close to their cut-off value.

## Abbreviations

ACA: anterior cerebral artery
CA: celiac artery
CI: confidence interval
CPAP: continuous positive airway pressure
CSA: confidence-step-analysis
LA/Ao-ratio: left-atrium-to-aortic-root-ratio
LVO/SVC-flow: left -ventricular -output-to-superior -vena -cava-flow - ratio
LVPEP/LVET: left-ventricular-preejection-period-to-ejection-time-ratio
PDA: patent ductus arteriosus
RepC: repeatability coefficient
RepI: repeatability index
RI: resistance index
SD: standard deviation
VLBW: very low birth weight
VTI_Ao: velocity time integral ascending Aorta
VTI_Pa: velocity time integral pulmonary artery
